# Quantitative Exposomics Targeting over 200 Toxicants and Key Biomarkers at the Picomolar Level

**DOI:** 10.1021/acs.est.5c04458

**Published:** 2025-10-10

**Authors:** Yunyun Gu, Max L. Feuerstein, Dillon T. Lloyd, Chirag J. Patel, Caroline H. Johnson, Benedikt Warth

**Affiliations:** † Department of Food Chemistry and Toxicology, Faculty of Chemistry, 27258 University of Vienna, Währinger Straße 42, Vienna 1090, Austria; ‡ Vienna Doctoral School of Chemistry, University of Vienna, Währinger Straße 42, Vienna 1090, Austria; § Exposome Austria, Research Infrastructure and National EIRENE Node, Vienna 1090, Austria; ∥ Department of Biomedical Informatics, 1811 Harvard Medical School, Boston, Massachusetts 02115, United States; ⊥ Department of Environmental Health Sciences, 50296 Yale School of Public Health, New Haven, Connecticut 06510, United States

**Keywords:** next-generation human biomonitoring, mass spectrometry, early life chemical exposure, exposome-wide association studies (ExWAS), public and environmental health

## Abstract

The exposome encompasses environmental exposures throughout life and significantly impacts health and disease. Exposure chemicals, present at trace levels, are frequently quantified using targeted LC–MS/MS. Many existing methods are limited to a narrow range of analyte classes or lack sufficient sensitivity for exposomic analyses, and applicability to large sample cohorts for exposome-wide association studies (ExWAS) remains to be demonstrated. Here, we present a scalable, fit-for-purpose next-generation human biomonitoring (HBM) workflow for analyzing >230 biomarkers in urine, plasma, and serum using solid-phase extraction in 96-well plates and LC–MS/MS. Moreover, a complementary conceptual framework for validation criteria of assays designed to analyze large panels of highly diverse compounds at trace levels is proposed. Method robustness was evaluated, demonstrating extraction recovery (60–130%), matrix effects (SSE, 60–130%), inter-/intraday precision (RSD <30%), and high sensitivity (limit of detection <0.1 ng/mL) for 59–80% of the analytes across the investigated biological matrices. To showcase the method’s applicability in epidemiological studies, 200 urine samples from pregnant women in a longitudinal pregnancy cohort were analyzed. More than 100 analytes including PFAS, drugs, air pollutants, pesticides, flame retardants, mycotoxins, industrial products, food processing contaminants, plastics-related chemicals, and phytotoxins, were detected, several for the first time in a U.S. urinary biomonitoring study. With its broad analyte coverage, ultimate sensitivity, robustness, and high sample throughput, this method meets the performance requirements for future large-scale ExWAS applications in public and personalized prevention research.

## Graphical abstract

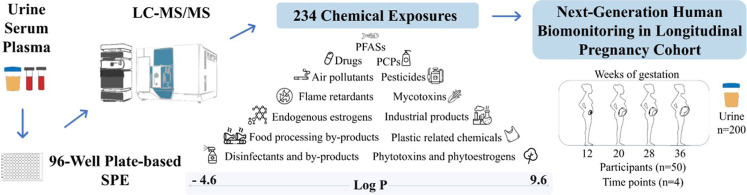

## Introduction

Individuals are exposed to a plethora of chemicals through dietary intake, inhalation, dermal contact, and other environmental sources. Exposomics comprehensively assesses these exposures and identifies potential links to adverse health effects, including chronic diseases or cancer.^1–3^ Liquid chromatography coupled to mass spectrometry, encompassing low-resolution mass spectrometry (LC–MS/MS) and high-resolution MS (LC-HRMS), is currently the most relevant technique in exposome-wide association studies (ExWAS) for targeted and nontargeted analysis (NTA).^4–6^ Targeted assays focus on quantifying predefined compounds with high sensitivity, selectivity, accuracy, and precision,^7,8^ while NTA aims to identify unknown chemicals and discover novel or unexpected exposures.^9^ Multianalyte targeted LC–MS/MS methods are ideal for quantifying various exposure chemicals at trace-levels due to favorable method sensitivity and robustness.^10,11^

Suitable sample preparation protocols are essential as sample matrix components can impact signal intensities, particularly for trace-level compounds.^12^ Various extraction protocols, including liquid extraction, protein precipitation (PPT), and solid phase extraction (SPE), are commonly used for diverse human matrices. PPT is widely used across omics studies due to its broad analyte coverage,^7,13,14^ whereas SPE is traditionally optimized for a smaller number of analytes classes. Broad analyte coverage is critical for multiclass analysis, but SPE protocols for diverse chemical classes remain rare due to varying analyte–sorbent interactions. Yet, SPE is increasingly popular in exposomics due to its efficient cleanup capabilities for complex samples.^15^ Recently, we developed a generic SPE protocol for 94 exposure compounds with diverse physicochemical properties and highlighted its applicability to targeted and nontargeted exposomics.^16^

To ensure suitable data quality, rigorous method validation is essential. The European Commission guideline No. 2021/808 provides systematic validation criteria (referred to as “EC criteria”) in terms of trueness, precision, and repeatability.^17^ However, the EC criteria and those of other guidelines may have limited applicability for exposome-scale LC–MS/MS approaches. Such methods can target hundreds of analytes in a single run, and workflow complexity requires a delicate balance between analytical coverage and method performance, e.g., due to a limited duty cycle that hampers data quality when targeting hundreds of analytes in a single run. Additionally, concentrations of most exposures are very low in human matrices, typically with concentrations in the pico- and nanomolar range. The EC criteria do not account for a higher number of diverse analytes and low concentrations. For instance, clear criteria for assessing precision for analytes below 120 μg/kg have not yet been established. Therefore, tailored validation criteria based on systematically evaluated empirical data may be a helpful addition to extend existing validation schemes for fit-for-purpose exposomic assays. Finally, applicability for large-scale studies should be tested to showcase the methods’ robustness, especially for populations with low-level exposures like pregnant women.^10,18–20^

In the presented work, an LC–MS/MS method was developed and validated for a high number of exposures, covering a wide range of physicochemical properties. For example, the analyte panel covered a log *P* range of −4.6 to 9.6. The method achieved detection limits down to the picomolar range and incorporated a 96-well plate-based SPE protocol for clean-up of human urine, plasma, and serum. Based on empirical data from relevant literature and generated within this study, a conceptual validation framework for exposome-scale methods is proposed as a potential complement to existing criteria. Fitness-for-purpose was assessed using the new approach and EC criteria for comparison purposes. The obtained validation results enabled the classification of analytes into fully quantitative, semiquantitative, or qualitative data. To assess the assay feasibility in a real-world exposure study, it was applied to 200 urine samples obtained from 50 pregnant US females during gestation (12^th^, 20^th^, 28^th^, and 36^th^ week).

## Materials and Methods

### Chemicals and Materials

The diverse analyte panel consisted of 234 compounds and was developed based on previous work.^7,12,21,22^ Furthermore, 58 additional endocrine-disrupting chemicals (EDCs) were selected based on relevant databases, i.e., compounds listed by the U.S. Environmental Protection Agency (EPA), European Human Biomonitoring Initiative (HBM4 EU), Comparative Toxicogenomics Database (CTD), and selected human biomonitoring (HBM) studies (see Table S1). In total, this panel covered 13 classes of compounds including perfluorinated alkylated substances (PFAS), medical drugs, personal care products, air pollutants, pesticides, flame retardants, mycotoxins, industrial products, food processing byproducts, plastics-related chemicals, disinfectants and byproducts, phytotoxins/-estrogens, and endogenous estrogens. Working stock solutions of all analytes (referred to as “STD mix”) were created by diluting individual stock solutions with acetonitrile.

The following isotope-labeled chemicals were used as internal standards: ^13^C_12_-bisphenol A, ^13^C_6_-butylparaben, ^13^C_15_-deoxynivalenol, ^2^H_3_-erythromycin, ^13^C_6_-ethylparaben, ^13^C_2_-monobutyl phthalate, ^13^C_6_-methylparaben, ^13^C_8_-perfluorooctanoic acid, ^13^C_8_-perfluorooctanesulfonic acid, ^13^C_6_-propylparaben, and ^13^C_18_-zearalenone. To account for variations in the SPE process, ^13^C_18_-zearalenone (100 ng/mL) was added before sample preparation, while the other internal standards (referred to as the “ISTD mix”) were added after SPE to track instrumental performance and matrix effects. Respective concentrations are provided in Table S2 and product numbers and suppliers are listed in Table S3. Oasis PRiME HLB 96-well plates (30 mg, 2 mL) were from Waters (Vienna). Further details regarding the preparation of stocks, mixtures, solvents, and spiking process are provided in the Supporting Information.

### Samples

Urine samples (*n* = 200) were collected from 50 pregnant women in Connecticut (U.S.) participating in the Yale Pregnancy Outcome Prediction Study (YPOPS). Participants self-collected samples at gestational weeks (GW) 12, 20, 28, and 36 during a single-day visit. These time points were selected since the 12^th^, 28^th^, and 36^th^ GWs mark the end of each trimester and the 20^th^ GW was when the routine fetal anatomy scan was performed. Urine was collected into sterile cups, kept on ice, and sent to a collection center, where 1.5 mL aliquots were transferred to cryovials, stored at −80 °C, and shipped on dry ice to the Global Exposomics and Biomonitoring Laboratory at the University of Vienna for analysis. Previously, these samples have been analyzed for biomarkers of mycotoxin exposure.^10^ The study was approved by the Yale University Human Investigation Committee (#1601017004), and samples were banked by the Yale University Reproductive Sciences (YURS) Biobank (HIC #1309012696).

Pooled urine, plasma, and serum samples were used for the method development and validation. The pooled urine was collected from a female volunteer following a two-day period of abstaining from foodstuff and beverages stored in plastic containers, phytoestrogen-rich foods, and cosmetics containing parabens.^12^ Pooled plasma was from Innovative Research (IPLAWBLIH-31982) (Novi, MI, USA), and serum was from Sigma-Aldrich (H4522, Sigma-Aldrich, Germany). Details of these commercial products are given in the Supporting Information. All samples were aliquoted and stored at −80 °C until analysis.

### Sample Preparation

A recently optimized SPE protocol utilizing 96-well plates was applied.^16^ In short, SPE plates were conditioned with methanol (MeOH) and water (H_2_O). Subsequently, 400 μL of sample was mixed with 4 μL of ^13^C_18_-zearalenone (100 ng/mL) and diluted with 396 μL of phosphate-buffered saline (PBS) before loading onto the SPE plates. Plates were washed with H_2_O and eluted with 2 × 200 μL of MeOH. Four microliters of the ISTD mix were added, and extracts were diluted with 396 μL of H_2_O, resulting in a total volume of 800 μL. Feasibility testing of the SPE protocol is visualized in Figure S1. In case of enzymatic hydrolysis tested during validation, a subset of urine samples was treated with β-glucuronidase and arylsulfatase (details provided in the Supporting Information section “Sample Preparation”, Enzyme Hydrolysis for Urine Prior to SPE Process).

### UHPLC–MS/MS

LC separation was performed using a generic reversed-phase method,^7,12^ with parameters presented in the Supporting Information and Table S4. Briefly, an Agilent 1290 Infinity II UPLC system was equipped with an Acquity HSS T3 column (1.8 μm, 2.1 × 100 mm) and a VanGuard precolumn (1.8 μm), both purchased from Waters. Retention times (RTs) for single compounds and potential RT shifts were determined by injecting standard solutions of single compounds or compound mixtures using concentrations of 0.5, 1, and 3 ng/mL.

During the optimization of the multiple reaction monitoring (MRM) method, transitions for targeted analytes were tuned individually, including precursor ion (Q1) and product ion (Q3) selection, optimization of entrance potential (EP), collision energy (CE), and collision cell exit potential (CXP). Values of Q1, Q3, EP, and CE were optimized on a QTRAP 7500 instrument (Sciex, Vienna) using method parameters of previously published methods as basis if available.^7,12,21,22^ MRM parameters for all additional analytes were optimized using direct infusion of standard solutions (5–10 ng/mL) with a syringe pump at a flow rate of 7 μL/min. Analytes were introduced either as mixtures or as individual solutions (Table S5). Poorly ionizing compounds were reoptimized individually. Two fragment ions were selected per compound as quantifier and qualifier ions. To ensure optimum data quality, dwell times were optimized for all targets to ensure enough data points per chromatographic peak. Fast polarity switching was used in scheduled MRM mode. More details, including ion source parameters and MRM transitions, are listed in Table S6.

### Quality Control, Quantification, and Statistics

A system suitability test was performed before and after each analytical sequence to track the instrument performance (Tables S7–S9). Multianalyte external calibration solutions were prepared in 50% MeOH in H_2_O at nine concentration levels (Table S10). Linear range and coefficient of determination (*R* ^2^) are reported in Table S11. Water was used for preparation of process blanks, and 50% MeOH was used as solvent blank to control possible background contamination and carryover. Three types of quality control (QC) samples were used including pooled samples (“nonspiked QC”) and pooled samples spiked after extraction (“postspiked QC”) and before extraction (“prespiked QC”). Furthermore, 11 isotopically labeled internal standards were used to track method performance during the analysis of 200 urine samples (for details, see Supporting Information). Sciex OS (version 4.1, Sciex, Vienna) was used to integrate peaks using the MQ4 algorithm and to build calibration curves for quantification (Supporting Information, Tables S10 and S11). Further data evaluation and visualization was done in Microsoft Excel (v16.0; Microsoft Office), PowerPoint (v16.0; Microsoft Office), Origin 2021b (v 9.8; OriginLab Corporation), and Inkscape (version 1.2.2; Inkscape). Concentration trends with gestation weeks were analyzed using a Linear Mixed-Effects Model (R lmer function, version 4.5.0). Details are reported in Supporting Information.

### In-House Validation

Full in-house validation was performed for urine and plasma matrices, while serum and hydrolyzed urine were subject to a more limited performance evaluation. Following the European Commission Decision (EC) No. 2021/808,^17^ performance parameters were evaluated over 3 days, including linearity, selectivity, matrix effects (signal suppression/enhancement, SSE), sensitivity (LOD and LOQ), trueness (extraction recovery, RE), intermediate precision (interday RSD, RSD_R_), and repeatability (intraday RSD, RSD_r_). Sample preparation, measurements, and data analysis were performed for each batch individually. Method linearity was evaluated by standards in solvent, and selectivity was tested by inspection of pooled urine and plasma samples for interferences. Matrix-matched calibration standards were prepared by spiking urine and plasma extracts at seven levels (Table S12). SSE (%) was calculated as the ratio of slopes of the matrix-matched and solvent calibration and was calculated for three batches.^23^ LODs and LOQs were determined by analyzing spiked urine and plasma samples at low concentrations as replicates (*m* = 9; see Table S13). Method-based LOD and LOQ were calculated based on signal-to-noise ratios of 1/3 and 1/10, respectively, if no background signal was present in the nonspiked matrix sample or, if a background signal was interfering, according to the EURACHEM guideline as 3 and 10 times the standard deviation of the measured concentrations divided by the square root of the number of replicates as described in the Supporting Information.^24^

RE was calculated based on determined concentrations in prespiked samples using matrix-matched calibration. RE was determined for two concentration levels, approximately 3 and 30 x LOQ (*n* = 3 each, see Table S14). Blank correction was performed by concentrations determined in nonspiked samples (*n* = 4) for RE calculation. RSD_R_ and RSD_r_ were determined as coefficient of variation (CV) of the RE within a single batch (*n* = 9) and within three validation batches.

To assess the method’s applicability across different human sample matrices, native and spiked pooled serum samples were extracted for evaluating trueness, precision, and matrix effects. The influence of enzymatic hydrolysis of urine samples on method performance was investigated by performing deglucuronidation and desulfation prior to SPE extraction and evaluating the trueness, repeatability, matrix effects, and sensitivity. The full validation details are provided in the Supporting Information, Tables S14 and S15.

### Validation Criteria for Trueness, Precision, and Repeatability Based on EC Guidelines

The EC criteria specify performance criteria for analytical method validation for a wide range of analyte concentrations. Acceptable ranges for trueness in terms of RE depend on the spiked concentrations. For concentrations >10 μg/kg, RE should be between 80% and 120%, 70–120% is acceptable for concentrations between 1 μg/kg and 10 μg/kg, whereas acceptable REs are in the range of 50–120% for concentrations <1 μg/kg. In analogy, acceptable values for RSD_R_ and RSD_r_, determined as CV of RE, are also concentration-dependent. Following the guideline, CVs <16% are required for concentrations >1000 μg/kg, and CV <22% is required for concentrations between 120 μg/kg and 1000 μg/kg. For concentrations <120 μg/kg, the guideline recommends minimizing CVs for both RSD_r_ and RSD_R_ to the lowest technically feasible level. To improve the comparability of mentioned concentration ranges with our data, we converted μg/kg to ng/mL. This conversion was performed by applying a multiplication factor of 1.0 g/mL, which approximates the densities of human urine and plasma (details in Table S16).

### Complementary Conceptual Framework for Validation Criteria in Exposome-Scale Studies

While current EC criteria provide the criteria for trueness, precision, and repeatability for analyte concentrations >120 ng/mL, a comprehensive framework for trace analysis in exposomic-scale investigations is currently missing. However, the high number of analytes per assay and the required ultimate method sensitivity in the pg-ng/mL range warrant tailored adjustment of analytical figures of merit. To address this challenge, we propose novel tailored validation criteria based on empirical data. Validation criteria for exposomics were developed using published validation data of exposomics assays for large analyte panels (i.e., >90 analytes) covering a wide range of physicochemical properties (i.e., covering a log *P* range of >5) and results from our experiments as a rational basis (see Table S17).^7,13^ Subsequently, the expected ranges for key parameters including RE, RSD_R_, and RSD_r_ values were derived as 5^th^ and 95^th^ percentiles of reported data and were tested as new criteria for assessment and comparison in exposomic-type LC–MS investigations. RSD_R_ and RSD_r_ values were derived as the 95^th^ percentile.

## Results and Discussion

### LC–MS Method Optimization

To ensure favorable method performance, RT windows and dwell times for all scheduled MRM transitions were optimized based on observed peak widths and RT shifts in biological matrices. Optimization of window width and dwell times is essential to balance the total cycle time, number of data points per peak, RT stability, and method sensitivity. Based on empirical observation of chromatographic peak widths, method development aimed for a total cycle time <850 ms, which could be achieved for the majority of all analytes (see Figure 1B). Due to unstable RTs in matrix samples, diethyl dithiophosphate, ibuprofen, perfluorodecanoic acid, and perfluorononanoic acid, as well as six phthalates were monitored during the entire runtime. After method optimization, the number of data points per peak was investigated in spiked samples (*n* = 3) to ensure sufficient data quality for quantification, with 91% of the analytes having >7 data points per peak (Table S18 and Figure S2), which can be regarded as sufficient for quantification.^25^

**Figure 1:**
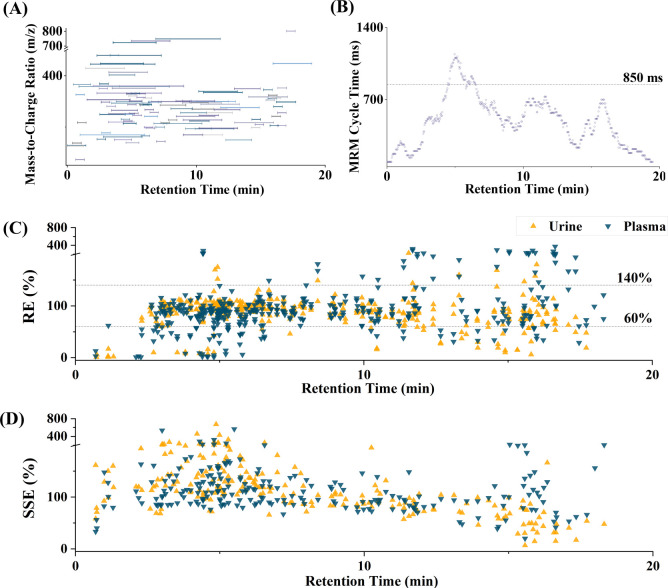
Scheduled Multiple Reaction Monitoring (MRM) windows (A) and MRM cycle time (ms, B) as a function of the chromatographic retention time (RT, min) of the optimized LC–MS/MS method. Extraction recovery RE (%, C) and signal suppression and enhancement SSE (%, D) in dependence of RTs for analytes in urine and plasma. Four analytes (2,5-dichlorophenol, xanthohumol, bisphenol M, and triclosan) are not shown in panel (C) due to high RE caused by interferences and/or matrix effects.

### Development of Tailored Validation Criteria for Exposomics

Several different guidelines for analytical method validation provide valuable frameworks and criteria for method validation in diverse applications areas. The choice of the used guideline and criteria typically depends on the specific research area. Among them, the EC guidelines provide a comprehensive framework for method validation for various application fields, yet several key challenges inherent to exposome-scale LC–MS/MS analysis are not addressed. Particularly, the methodological complexity and sensitivity requirements necessary for detecting a large range of diverse analyte mixtures at trace levels in a single assay are not fully addressed. To fill this gap, we developed a tailored framework for validation criteria for exposomics based on published data sets of exposomics-scale LC–MS/MS methods and our own data set presented as part of this work. Therefore, we included methods quantifying >90 analytes from different analyte classes with a broad log *P* range >5 (Table S19). These proposed novel in-house validation criteria for exposomics-scale multianalyte assays were derived from empirical data using the 5th and 95th percentiles of the compiled data sets. Figure 2A–C show the distribution of RE and RSD reported in two published methods^7,13^ and results from the study at hand, indicating largely comparable results between the included data sets. The 5th and 95th percentiles of RE were calculated as 42% and 134%, respectively, highlighting certain limitations in terms of analyte recovery for currently used LC–MS workflows in the case of sets of extremely diverse analytes at trace concentrations. These empirically determined limits were subsequently proposed as the acceptable range for RE in quantitative exposomics (Figure 2A), whereas the 95th percentile was used as the upper limit for assay repeatability (RSD_r_ <37%) and intermediate precision (RSD_R_ <42%) (Figure 2B,C). These custom validation criteria were then used to complement the EC criteria for the relevant concentration range in exposome research (i.e., <1 ng/mL) and were integrated into our proposed complementary method validation framework (see Figure 2D and Table S16). Figure 2 illustrates a schematic representation of the criteria provided in the EC guidelines and proposed new data-derived criteria for exposomics including relevant concentration ranges. Furthermore, a simple three-level scheme is suggested to categorize quantitation confidence for the targeted analytes based on validation results: quantitative data is reported as data quality category A, semiquantitative data as category B, and qualitative data as category C. Detailed definitions for A, B, and C are described below (section Classification of Data Quality in Quantitative Exposomics). We see this framework as a starting point to spark a discussion within the field concerning “fit-for-purpose” approaches rather than a finalized set of criteria. It is not intended to replace established standard guidelines but to complement them.

**Figure 2:**
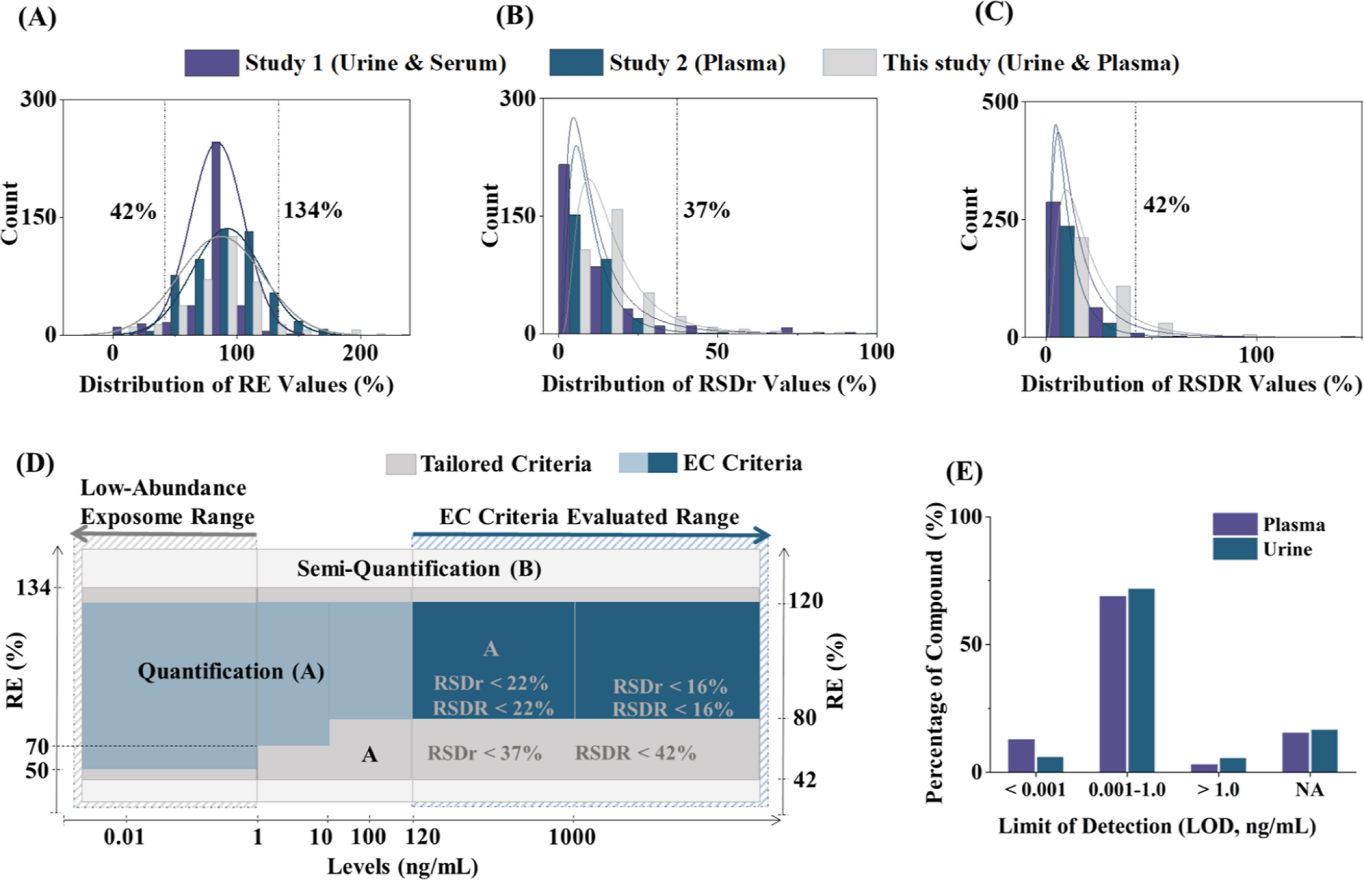
Distribution of extraction recovery RE (%, A), repeatability RSD_r_ (%, B), and precision RSD_R_ (%, C) reported in two published exposome-scale targeted LC–MS/MS studies^7,13^ and data generated in this study. Empirical data was used to derive tailored validation criteria for exposome-type method validation to complement and extend EC criteria (EC No. 2021/808)^17^ for low-abundance analyte concentrations in multianalyte assays (D). Panel (E) shows the distribution of the method-based LOD values in the present study. NA, not applicable with explanations reported in Table S20.

### Results of the In-House Validation

Excellent method linearity was achieved, with 88% of compounds achieving *R* ^2^ values exceeding 0.99 in pure solvents (Table S11). Sensitivity was also excellent for the majority of analytes with LOD values <1 ng/mL observed for 73% of compounds in urine and 74% in plasma. Approximately 10% of the analytes showed exceptionally low LODs (<0.001 ng/mL) in both sample matrices (Figure 2E). For approximately 70% of the analytes, RE values were in the range of 42–134% in both sample matrices (Figure 3A), and 80% and 77% of the analytes showed RSD_r_ <37% in plasma and urine, respectively. Notably, 63% displayed RSD_r_ values below 20% (Figure 3B). RSD_R_ was determined, with 67% of all compounds showing RSD_R_ <37% and 38% and 57% of compounds showing RSD_R_ <20% in plasma and urine, respectively (Figure 3C). Matrix effects were evaluated, and effective sample cleanup with SPE was demonstrated for many analytes, as previously reported for a smaller subset of the analyte panel.^16^ In total, 61% of the analytes in urine and 70% in plasma showed SSE within 60–140% (Figure 3D). For compounds with SSE values outside the 60–140% range in both, urine and plasma, distinct trends emerged: some polar compounds (including specific drugs, mycotoxins, pesticides, and phytotoxins and phytoestrogens), typically with log *P* below 3.0, exhibited matrix enhancement (SSE > 140%), while some nonpolar chemicals (e.g., personal care products), primarily with log *P* above 3.0, showed matrix suppression (SSE < 60%). Full validation results are provided in Tables S14 and S20. For serum and hydrolyzed urine, validation results are presented in the Supporting Information (Figure S3 and Tables S14 and S20).

**Figure 3:**
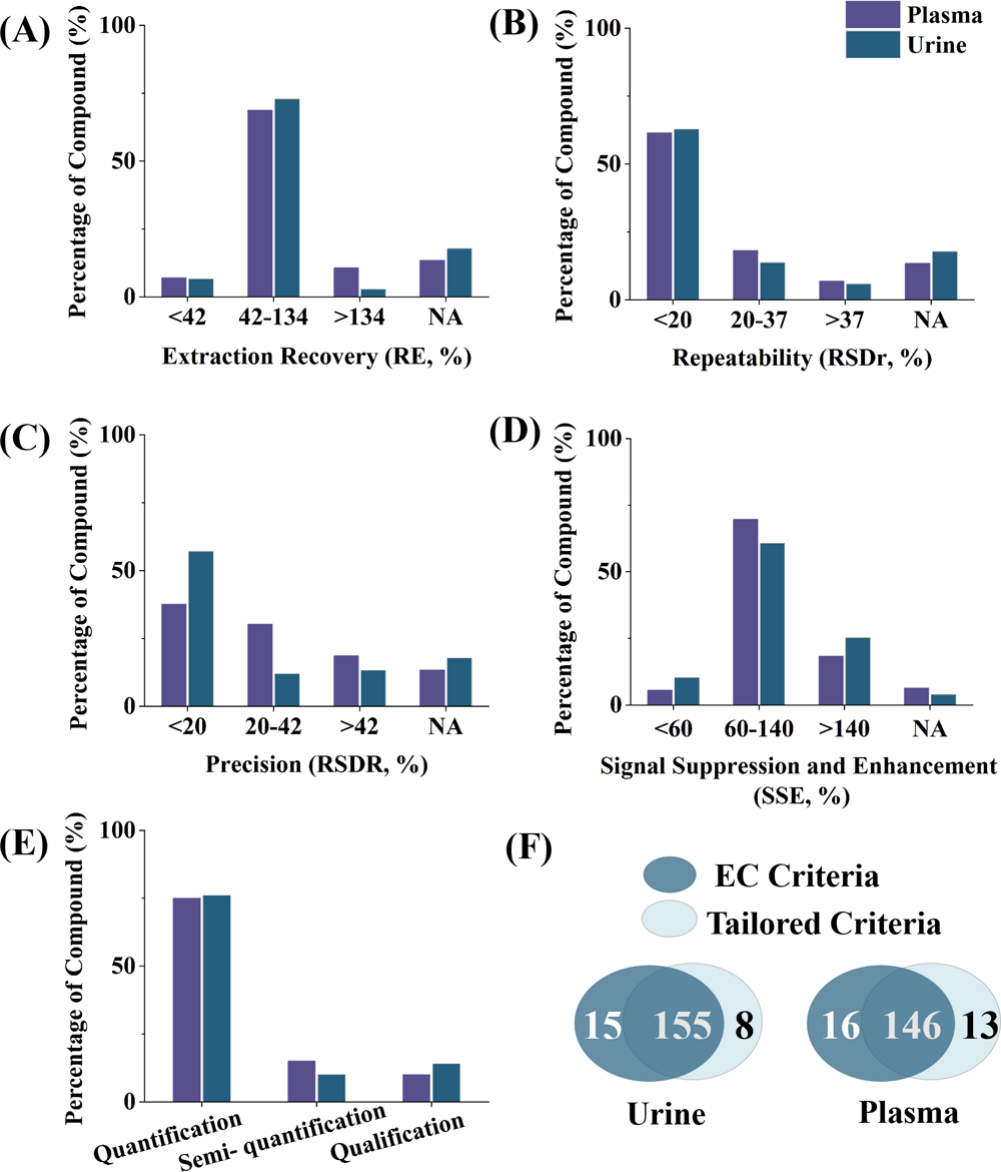
Results of the in-house method validation: analytes with RE (%, A), RSD_r_ (%, B), RSD_R_ (%, C), and SSE (%, D) within the range required for quantitative exposomics. Classified validation results following EC criteria^17^ and proposed tailored criteria using three categories: quantification, semiquantification, and qualification (E). Number of analytes classified as “quantification” for at least one spiking level according to the EC criteria and/or the tailored criteria (F). NA, and applicable data with explanations reported in Tables S14 and S20.

### Classification of Data Quality in Quantitative Exposomics

To be in line with the EC criteria, analytes need to meet the following requirements for at least one spiking level to be reported as quantitative data (category A): (1) RE within 50–120% for concentrations ≤1 ng/mL, between 70% and 120% for concentrations between 1–10 ng/mL, and 80–120% for concentrations between 10 and 120 ng/mL or higher. (2) RSD_R_ and RSD_r_ ≤ 22% are required for concentrations within 120–1000 ng/mL, RSD_R_ and RSD_r_ ≤ 16% for concentrations >1000 ng/mL, whereas no clear limit is defined for RSD_R_ and RSD_r_ for compounds at trace and ultratrace levels (see Figure 2D). Using the proposed tailored criteria, data was reported as quantitative if the following requirements were met for at least one spiking level: (1) RE in the range of 42–134% and (2) RSD_r_ ≤37% and RSD_R_ ≤42% (Figure 2D). To implement a clear scheme for reporting quantitative, semiquantitative, and qualitative data in exposomics, two additional categories were introduced for the classification of the data quality: data was classified as semiquantitative (category B) if either RE or RSD values failed to meet quality criteria for quantitative data described above. Qualitative data (category C) was reported for compounds with stable RT, but accuracy and repeatability could not be evaluated due to poor sensitivity or high background. More details are listed in Table S14.

Figure 3F illustrates the number of quantitative analytes meeting the EC criteria and/or the tailored criteria. Using the EC criteria, 170 analytes were classified as “quantitative” in urine, whereas using the tailored criteria results in 163 analytes. In plasma, the EC criteria resulted in 162 quantifiable analytes, compared to 159 using our tailored criteria. Noteworthily, most analytes (155 in urine and 146 in plasma) met the quantification requirements of both validation schemes. A subset of compounds (15 in urine and 16 in plasma) met quantification category A under the EC criteria, yet were classified as category B when using the tailored criteria. This difference reflects the EC criteria’s greater tolerance for poor precision and repeatability at extremely low analyte concentrations. In contrast, another subset of analytes (8 in urine and 13 in plasma) was classified as semiquantitative (category B) using EC criteria but as quantitative (category A) using the tailored classification scheme due to a higher tolerance in regards of RE. Here, the tailored criteria allow higher tolerance for trueness, due to the complexity of large-scale exposome-scale assay. This takes into consideration that correction for “trueness” (i.e., lower RE) is often possible if the method’s precision is acceptable and a suitable correction approach (e.g., using internal standards or quality control samples) is used. Given the complementary nature of both approaches and the complex nature of large-scale multianalyte LC–MS/MS assays, compounds meeting the quality criteria of either of the validation schemes were considered as suitable for quantification. In total, 76% and 75% of all analytes met the requirements for quantification (category A) in urine and plasma, respectively. Another 10% and 15% of all analytes met the requirements for semiquantification (category B), whereas the remaining 14% and 10% can only be reported as qualitative data (i.e., detected and nondetected, category C) in urine and plasma, respectively (see Figure 3E).

### Benchmarking Coverage, Sensitivity, and Throughput

The presented validated exposomics workflow allows analyzing diverse exposure chemicals covering a wide range of polarities and concentrations. Method performance and coverage were compared to three relevant published LC–MS/MS protocols for benchmarking (Table S17). Noteworthily, the presented method covers the widest range of analyte polarities and achieved comparable sensitivity for the majority of analytes (Figure 4). Especially, the fraction of extremely sensitive analytes (LOQ < 0.0033 ng/mL) was approximately two times higher than in previously published studies (see Figure 4B).^7,13,26^

**Figure 4:**
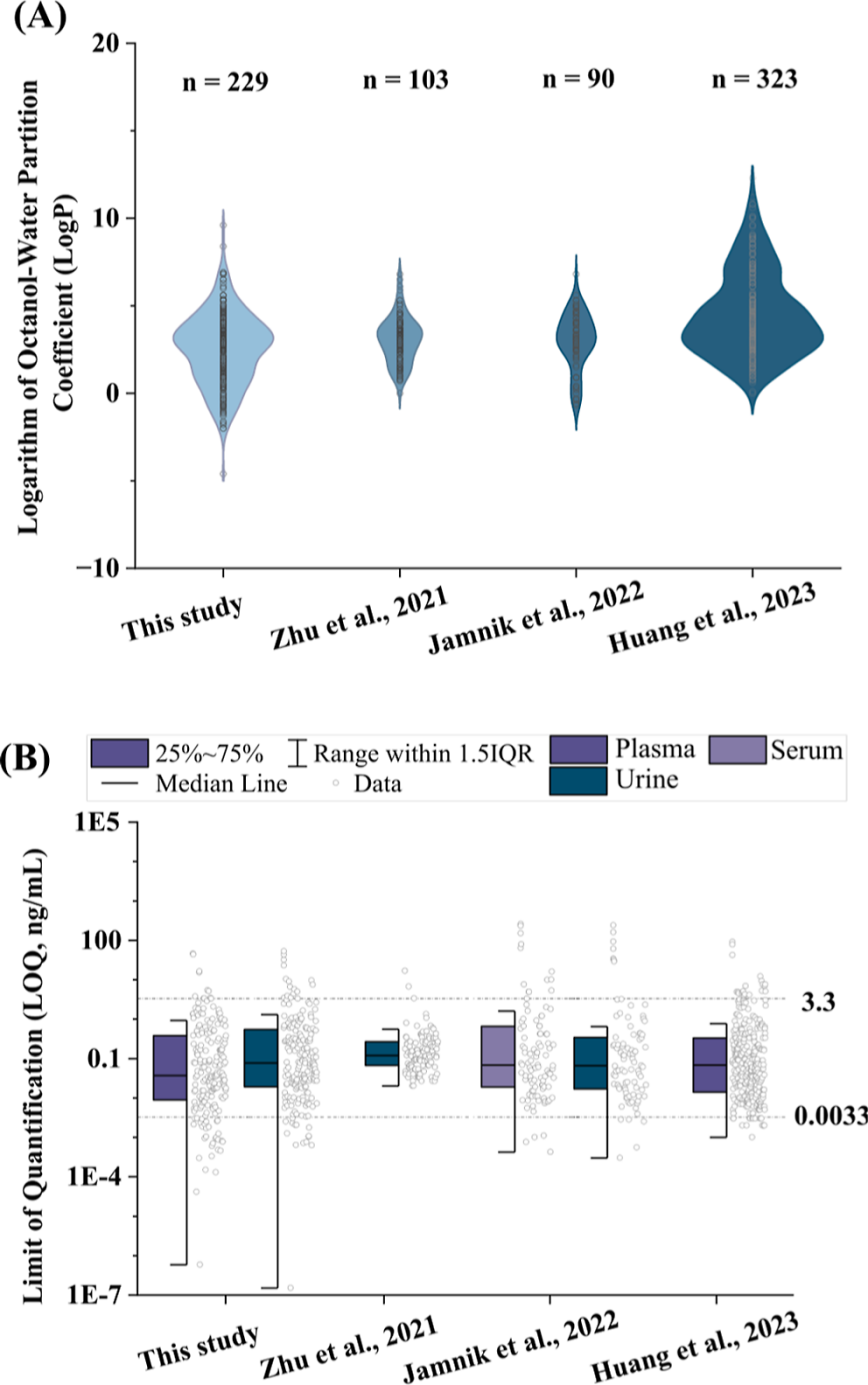
(A) Coverage of the chemical space of multianalyte exposomics assays in terms of logarithm of octanol–water partition coefficient (log *P*) values for analytes in this study (*n* = 229) and three published papers: Zhu et al. (2021; *n* = 103)^26^, Jamnik et al. (2022; *n* = 90)^7^, and Huang et al. (2023; *n* = 323) ^13^. (B) Limit of quantification (LOQ, ng/mL) for analytes in plasma (*n* = 209) and urine (*n* = 206) in this study, in urine (*n* = 114) in Zhu et al. (2021)^26^, serum (*n* = 92) and urine (*n* = 87) in Jamnik et al. (2022)^7^, and plasma (*n* = 295) in Huang et al. (2023)^13^. Note: Due to the lack of reported LOQ values in Zhu et al. (2021)^26^, we estimated LOQs as 3.33 x the reported LODs in (B). Log *P* values listed in PubChem (https://pubchem.ncbi.nlm.nih.gov) were used for panel (A). IQR, interquartile range.

While Huang et al. (2023)^13^ presented a method with more analytes, the presented workflow (*n* = 234) offers the most diverse set in terms of analyte log *P*. This is a critical metric for demonstrating sufficient coverage and scalability potential for exposomics applications, highlighting its competitive edge among state-of-the-art LC–MS/MS-based exposomics workflows (Figure 4A).^7,13,26^ Assay sensitivity was benchmarked by comparing method LOQs against relevant literature, focusing on analytes with good sensitivity (LOQs < 3.3 ng/mL). While the overall number of such analytes was comparable to other assays, our method demonstrated outstanding sensitivity for 6–13% of compounds, achieving LOQs < 0.0033 ng/mL in both urine and plasma. In contrast, other published work reported comparable LOQs for only 4–7% of analytes (Figure 4B and Table S17).

The applicability of the developed method for high-throughput exposomics was assessed and compared to other protocols in terms of sample requirements, solvent consumption, and total analysis time (Table S21). Using the SPE method presented herein, 1.4 mL of solvents are required per sample, compared to 0.8 mL estimated for a previously published PPT protocol.^7^ Similar calculations were performed for other published works, resulting in even higher solvent consumption of approximately 6 mL per sample calculated for the sample preparation protocol of Huang et al. (2023).^13^ While these estimations include some uncertainties, this further highlights the high efficiency of our workflow, which would result in a theoretical reduction of 4.6 L solvents calculated for the extraction of 1000 samples. Finally, sample preparation in 96-well plates is time efficient. It was estimated that the total required laboratory time of ∼20 h for sample preparation 1000 samples including SPE cleanup is sufficient, whereas the same number of samples would need ∼175 h of laboratory work using a traditional PPT protocols in Eppendorf tubes.^7,16^ This estimate intentionally excludes factors such as injection time and data processing, as these are highly variable and depend on the specific instruments and software used.

### Real-Life Exposure Patterns in Pregnant Women from the YPOPS Cohort

To demonstrate favorable sensitivity and general performance of the new workflow and its applicability to real-life research questions, 200 urine samples of women (*n* = 50) from a U.S. pregnancy cohort were analyzed. Pregnant women present a highly vulnerable population group, and expected exposure levels are typically lower than in the average population. Yet, 109 out of 234 target analytes were detected, with 25 exposure compounds present with very high detection frequencies (DF) above 70% (Figure 5). The detected and quantified biomarkers of exposure included chemicals from various exposure routes, application fields, and compound classes including antibiotics, mycotoxins, medical drugs, pesticides, personal care products, phytotoxins, phytoestrogens, plasticizers, and PFAS chemicals.

**Figure 5:**
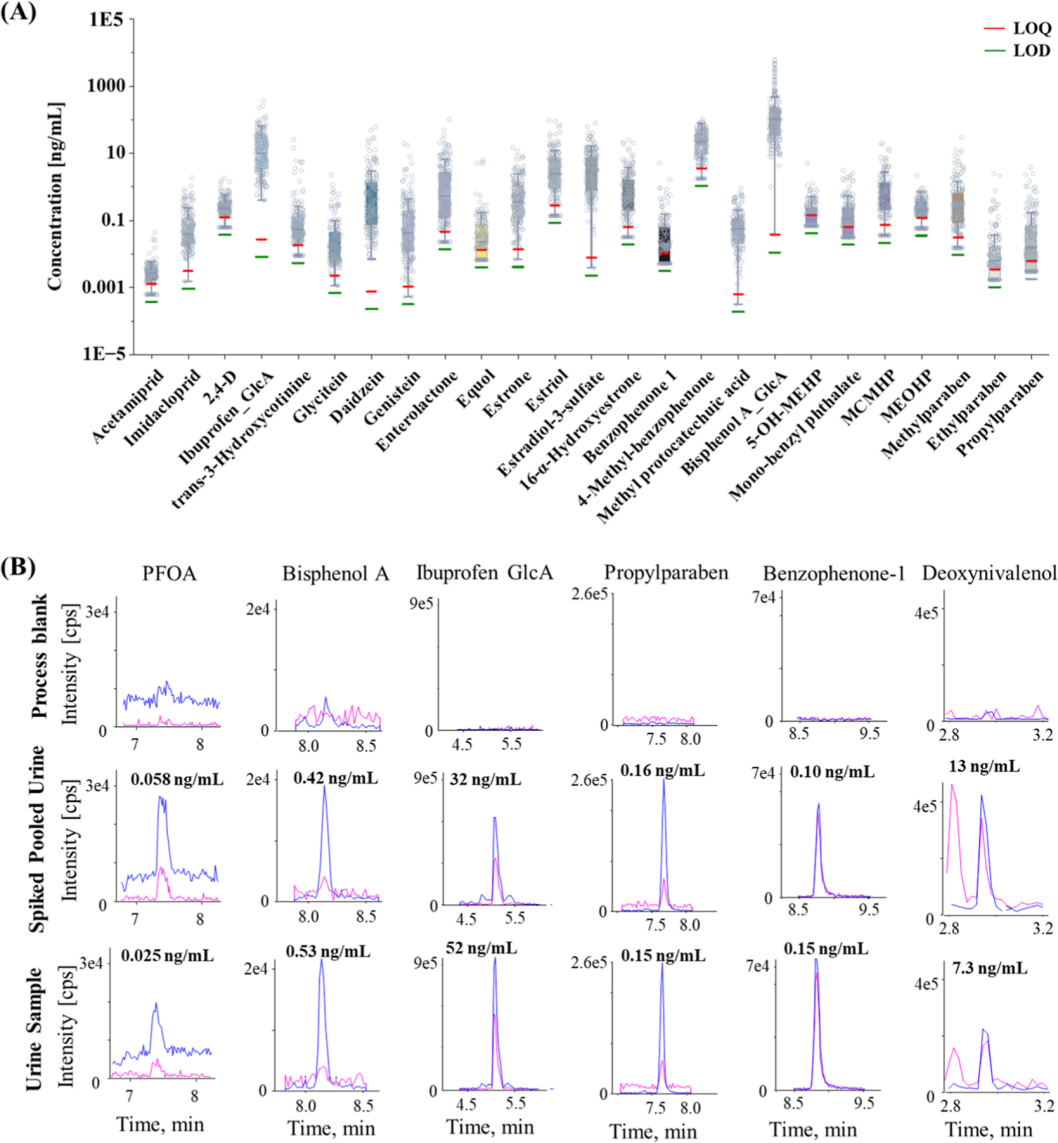
(A) Concentrations of 25 frequently detected compounds (DF > 70%) in urine samples from pregnant women in the YPOPS cohort. (B) Representative chromatograms of compounds in process blank, spiked pooled urine, and a detected sample from the cohort. Note: Concentrations below the limit of quantification (LOQ) were imputed with LOQ/2. 2,4-D, 2,4 dichlorphenoxyacetic acid; GlcA, glucuronide; 5-OH-MEHP, mono-(2-ethyl-5-hydroxyhexyl) phthalate; MCMHP, mono-[(2-carboxymethyl) hexyl] phthalate; MEOHP, mono-(2-ethyl-5-oxohexyl) phthalate.

Examples for Selected Toxicants are Briefly Discussed Below. The full data set including the classification of the data quality based on the obtained validation data is presented in Table S22. This table provides an overview of all of the compounds. A more detailed breakdown of the results according to quantitation categories are reported in Tables S22a–c: S22a for quantified compounds; S22b for semiquantified analytes; and S22c for qualified compounds in cohort samples. The detailed results are listed in Table S23. For instance, urinary cotinine, a biomarker for active or passive cigarette smoke, was determined in 46% of the samples (median concentration < 0.15 ng/mL, range < 0.15 ng/mL – 23 ng/mL), demonstrating again the high sensitivity (LOD, 0.03 ng/mL). In analogy, *trans*-3-hydroxycotinine, a phase-I-metabolite of cotinine, was quantified in 85% of the samples at a lower median concentration (median 0.053 ng/mL, range < 0.018 ng/mL – 25 ng/mL). Importantly, these levels typically indicate environmental/passive exposure to cigarette smoke rather than active smoking.

Several PFAS chemicals were detected including perfluorooctanoic acid (PFOA; median 0.024 ng/mL, range < 0.0071 ng/mL – 0.12 ng/mL) in 66% of the samples. Ten out of 12 monitored bisphenol derivatives or metabolites were quantified in the sample set. As expected, the BPA phase-II metabolite, BPA-glucuronide, was the most frequently detected analyte of this class. However, data quality allows only qualitative assessment of this analyte in the presented data set (data quality category C—qualitative results). The parent molecule BPA was less frequently detected due to a higher LOD value. BPF was also frequently detected in 62% of the samples (median 0.44 ng/mL, range <0.12 ng/mL – 1.5 ng/mL).

The pesticides acetamiprid, imidacloprid (Figure 5), the pesticide metabolite 2-isopropyl-4-methyl-6-hydroxypyrimidine (IMPY), and 2,4-dichlorophenoxyacetic acid (2,4-D) were detected in most samples. The results were compared to other published data sets of pesticide exposure in pregnant women from Asia and Europe. For acetamiprid and imidacloprid, DFs of 89% and 98% were observed in our study, with concentration levels ranging between <0.0012 – 0.044 ng/mL and 0.0031 – 1.8 ng/mL, respectively. Lower DFs (18% and 26%) and comparable urinary concentrations (<0.0025 ng/mL – 1.3 ng/mL and <0.025 ng/mL – 0.95 ng/mL) were reported for 617 urine samples collected from 62 pregnant women in Japan.^27^ Similarly, IMPY, which is a key metabolite of the organophosphate pesticide diazinon, was detected at concentration levels between <0.023 ng/mL and 2.1 ng/mL (DF = 25%) in the present study. In a study from Spain, IMPY was present at concentrations between <1.6 ng/mL and 744.2 ng/mL and was detected in 12% of urine samples from pregnant women (*n* = 573) in Valencia.^28^ 2,4-D was detected with a concentration range of < 0.12 ng/mL – 1.8 ng/mL for most samples (DF = 99%) in our study. In data sets of the National Health and Nutrition Examination Survey (NHANES) from 1999 to 2014, 2,4-D was reported at concentrations of 0.010 ng/mL – 18 ng/mL with lower DF (75%) for urine samples collected from pregnant women (*n* = 398) in the United States.^29^

The data obtained from this high-performance multianalyte assay also demonstrated proper performance when comparing it to a more tailored HBM assay covering multiple mycotoxins that was applied to the same set of sample before.^10^ The most concentrated and prevalent fungal toxin, deoxynivalenol (DON) was determined in 42% of the samples with a median concentration of <3.5 ng/mL (Table S22), notably in its native form. The median in the tailored HBM assay that measured total deoxynivalenol after enzymatic deconjugation of glucuronide and sulfate conjugates was reported as 23 ng/mL (DF = 99%, 0.95 ng/mL – 436 ng/mL).^10^ Given the fact that the vast majority of deoxynivalenol is present conjugated in human urine,^30,31^ this demonstrated comparable performance of the new assay for the assessment of DON exposure. The same is true for the *Alternaria* toxin alternariol (DF = 14%), in line with the quantitative results in a cohort of primary school children in Austria (DF = 8%).^32^ For citrinin, a nephrotoxic mycotoxin, the sensitivity of the presented assay was even enhanced so that a low number of samples indicated exposure while the previously published, tailored HBM assay was unable to detect this food contaminant in urine. The same was true of enniatin B, a Fusarium toxin, which was quantified in one sample (0.0064 ng/mL). Beauvericin was detected (DF = 2%) in human urine biomonitoring for the first time to the best of our knowledge (Table S22).

In addition, many other natural toxins and bioactives have been identified and quantified. Some of them are detected in the urine from U.S. citizens for the first time to the best of our knowledge, while others, especially those occurring at higher concentrations, have been reported before.^33,34^ The first-time detection of compounds in urine from U.S. citizens includes aristolactam I, a nephrotoxic plant toxin (Table S23).

The exposome is defined as the totality of exposure to the (chemical) environment over the lifespan, and longitudinal studies are of special relevance. In the investigated sample cohort of the YPOPS study, 50 pregnant women were sampled at four time points during pregnancy. Table S24 reports statistically significant trends through gestation weeks. Eleven compounds, comprising nine endogenous hormones, the insecticide acetamiprid, and the phytoestrogen daidzein, showed increasing trends (*p* < 0.05). Conversely, ethylparaben and four plasticizers, including monobutyl phthalate, monobenzyl phthalate, bisphenol F, and bisphenol S, significantly decreased over the same period (Figure 6 and Table S24). However, due to the mild changes and the limited sample number, these results need to be interpreted with caution.

**Figure 6:**
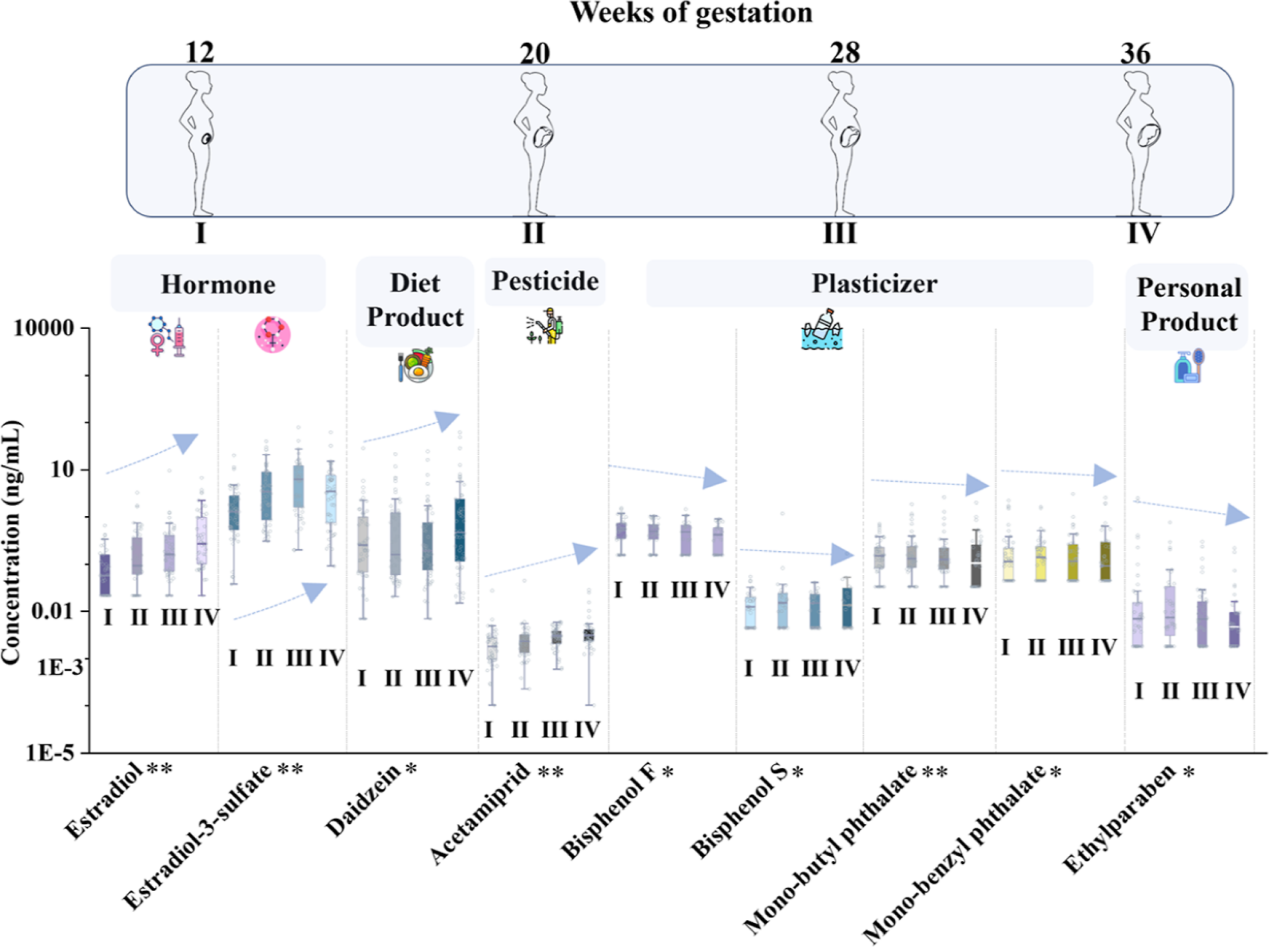
Longitudinal concentration profiles of frequently detected compounds (DF > 50%) in urine samples obtained from 50 pregnant females with significant trends (*p* < 0.05) across four gestational periods in the Yale Pregnancy Outcome Prediction Study (YPOPS). Here, concentrations below the limit of quantification (LOQ) were replaced with a value of LOQ/2, and concentrations below limit of detection (LOD) are not shown. *p < 0.05; **p < 0.01. The indicated trends need to be interpreted with caution, and it needs to be considered that other exposures were stable throughout pregnancy.

### Limitations

While the robustness and sensitivity as well as time and cost-efficiency of the developed workflow were clearly demonstrated, several limitations remain. Although the presented analyte panel was well designed and aims for high diversity and representativeness, still only a limited subset of the chemical exposome can be covered in a single assay. For a comprehensive assessment of the human exposome, even wider chemical coverage, combinations of complementary methods, or more human matrices are required. Furthermore, several very polar compounds (8%) have been lost during the SPE process due to weak retention with the sorbent, and substantial matrix effects were observed for some other analytes (6%). Combinations of different orthogonal sample preparation and analyte separation approaches might address these limitations, yet this is time-consuming and costly. Clearly, broad coverage exposome-type assays need to find a well-balanced compromise between analyte coverage, efficient sample cleanup, assay sensitivity, and time requirements. The overall performance of the method in the real-life case study yielded highly information-rich exposure patterns, indicating that the chosen pragmatic approach is fit-for-purpose. While the insights on longitudinal exposure patterns in pregnant U.S. women are of high novelty, it needs to be noted that the cohort may not be representative for other exposomic applications.

Traditionally used criteria for analytical method validation show limited applicability for exposome research, because of the required high sensitivity and method complexity. Our newly established validation criteria aim to propose a complementary, conceptionally novel, and evidence-based framework to systematically extend established guidelines for analytical method validation to meet the requirements of quantitative exposomics. This is relevant to ensure comparable reporting standards and method performance. Yet, our proposed tailored validation criteria rely on data derived from only a small number of published exposome-scale studies (*n* = 3) due to limited availability of fully validated exposome-scale LC–MS/MS assays. Further integration of additional data sets would be essential before providing a comprehensive framework for method validation in exposomics-scale HBM.

### Implications

In summary, a high-performance workflow for exposome analysis characterized by broad chemical coverage and good method sensitivity across diverse human matrices was developed. The analytical pipeline was validated based on existing guidelines and using a conceptionally new and tailored framework of validation criteria designed for exposomics. This is necessary to face typically observed challenges in exposomics such as broad exposome-scale analyte coverage and compounds present at trace concentrations. Method performance was characterized and offers a robust foundation for quantifying numerous priority exposures in population-scale studies. The versatile assay extends analyte coverage beyond traditional food and environmental toxicants and includes biotransformation products, medical drugs, and microbiome-related compounds besides typically monitored toxins and contaminants of emerging concern. Laboratory sample throughput presents one of the critical bottlenecks in large-scale exposomics, and we reduced the required laboratory work by approximately a factor of 10 using SPE in 96-well plates. The scalability of the method was demonstrated and now offers the required capacity to perform large-scale population-based studies for exposome-wide association studies (ExWAS). Such large-scale studies are essential to enhancing our understanding of the chemical exposome, which is needed to improve disease prevention in public health and personalized medicine. Due to the high method sensitivity and sample throughput, the presented validated workflow demonstrated its fitness for purpose to increase our understanding of the chemical exposome and will be used in large-scale population studies. Such environmental health studies will be crucial for unraveling the relationships between (co)­exposure to diverse exposure chemicals and human disease in the future.

## Supplementary Material





## Notes

Detailed descriptions on materials, sample preparation, LC–MS/MS analysis, in-house validation, and assessment for efficacy and environmental impact, results and discussion, and supplementary figures (PDF)
Detailed tables of standards, classifications, concentrations of standards, information on LC–MS/MS parameters, validation results, and detailed results from YPOPS urine samples (XLSX)
