# Supplementary material for: Quantitative Exposomics Targeting over 200 Toxicants and Key Biomarkers at the Picomolar Level
Source: Environ Sci Technol. 2025 Oct 10;59(41):21818–29. doi: 10.1021/acs.est.5c04458 (PMC12550810; doi:10.1021/acs.est.5c04458)
Supplement: Supplementary file 1 [file es5c04458_si_001.pdf]

<u>Supporting Information</u>

Quantitative exposomics targeting over 200 toxicants and key biomarkers at the picomolar level

Yunyun Gu[a,b], Max L. Feuerstein[a,c], Dillon T. Lloyd[d], Chirag J. Patel[d], Caroline H. Johnson[e], Benedikt Warth[a,b,c*]

[a]Department of Food Chemistry and Toxicology, Faculty of Chemistry, University of Vienna, Währinger Straße 42, Vienna, 1090, Austria

[b]Vienna Doctoral School of Chemistry, University of Vienna, Währinger Straße 42, Vienna, 1090, Austria

[c]Exposome Austria, Research Infrastructure and National EIRENE Node, Währinger Straße 42, Vienna, 1090, Austria

[d]Department of Biomedical Informatics, Harvard Medical School, Boston, MA 02115, United States

[e]Department of Environmental Health Sciences, Yale School of Public Health, New Haven, CT 06510, United States

Corresponding author

*Benedikt Warth, E-mail: benedikt.warth@univie.ac.at, Phone: +43 1 4277 70806

Department of Food Chemistry and Toxicology, Faculty of Chemistry, University of Vienna, Währinger Straße 38, Vienna, 1090, Austria

Table of Contents

Materials 2

Sample Preparation 3

UPLC-MS/MS Analysis 4

In-house Validation 5

Assessment for Efficacy and Environmental Impact 7

Results and Discussion 7

Supplementary Figures 8

Reference 11

Materials

Method development and optimization involved the evaluation of 234 chemicals. This comprehensive panel of exposure compounds, based on previous work, expanded from a workflow focusing on mycotoxins (Braun et al., 2022), a multi-endocrine disrupting chemical (EDC) panel (Preindl et al., 2019) to a full-scale Next-Generation human biomonitoring (HBM) method. This expanded method (Jamnik et al., 2022) encompassed environmental and food toxicants across diverse chemical classes and was further scaled up to include antibiotics, veterinary drugs and pesticides (Hossain et al., 2024). As a result, the final panel of 234 analytes consisted of 176 well established analytes (Table S19) from our previous work, and 58 additional endocrine disrupting chemicals (EDCs, Table S1) listed in relevant priority lists and databases, including the U.S. Environmental Protection Agency (EPA), the European Human Biomonitoring Initiative (HBM4EU), Comparative Toxicogenomics Database (CTD), and current human biomonitoring studies (see Table S1).

This includes the four disinfectants 2,4,5-trichlorophenol, 2,5-dichlorophenol, 2- and 4-phenylphenol, which are listed in the EPA's Endocrine Disruptor Screening Program (EPA|EDSP, www.epa.gov/comptox-tools). Previous studies reported low levels of these analytes in urine samples of adolescents and children with median concentrations of 0.02–4.64 ng/mL (Frederiksen et al., 2020; Guo et al., 2019). Ten additional personal care product-related compounds were selected based on EPA|EDSP, EPA's Ecotoxicology knowledgebase (EPA|ECOTOX) and the Comparative Toxicogenomics Database (CTD). Furthermore, 23 plastic related compounds, five bisphenols and 18 phthalates, were selected based on the EPA|EDSP, EPA|ECOTOX, Toxicity Values database (EPA|ToxValDB), EPA's Multimedia Monitoring Database (EPA|MMDB), and Chemicals of Emerging Concern (CEC) listed in the HBM4EU database (HBM4EU|CECscreen). These chemicals were previously reported in urine and serum from pregnant women, children and adolescents with median concentrations in the range of 0.09 – 46 ng/mL (Caballero-Casero et al., 2021; Frederiksen et al., 2020; Frigerio et al., 2020; Guo et al., 2021; Gys et al., 2021; Jala et al., 2022; Varghese et al., 2022). Five flame retardants, tri-n-butyl phosphate, triphenyl phosphate, tris(2-chlorethyl)phosphate, bis(1,3-dichloro-2-propyl)phosphate, and bis(1-chloro-2-propyl)phosphate, were selected based on EPA|EDSP and EPA|ECOTOX lists and due to reports in human urine and blood samples (Gao et al., 2020; Li et al., 2020; Siddique et al., 2020). Finally, five PFAS substances (perfluorobutane sulfonic acid, perfluorodecanoic acid, perfluorohexanoic acid, perfluorononanoic acid, and perfluoroundecanoic acid), three drugs (duloxetine, tramadol and venlafaxine), three bioactive phytochemicals (chrysin, chalcone, and kaempferol), and five pesticides (glyphosate, vinclozolin, propiconazole, tebuconazole, and metribuzin) listed in EPA ECOTOX or EPAEDSP database were added.

Stock solutions (1- 10 mg/mL) for 176 compounds were prepared in acetonitrile (ACN). The 58 EDCs were prepared in methanol (MeOH) or ACN, with exceptions for chrysin (formic acid/dimethyl sulfoxide/methanol, FA/DMSO/MeOH, 5/200/1000, v/v/v) and glyphosate (50% MeOH in water). EDC stock concentrations ranged from 1 to 10 mg/mL. A spiking mixture of the 234 chemicals was created by diluting all stocks with ACN, which will be referred to as "STD mix" in the SI and manuscript. A mixture of ten isotope-labeled internal standards ($^{13}C_{12}$-bisphenol A, $^{13}C_6$-butylparaben, $^{13}C_{15}$-deoxynivalenol, $^{2}H_3$-erythromycin, $^{13}C_6$-ethylparaben, $^{13}C_2$-mono-butyl phthalate, $^{13}C_6$-methylparaben, $^{13}C_8$-perfluorooctanoic acid, $^{13}C_8$-perfluorooctanesulfonic acid, and $^{13}C_6$-propylparaben) was prepared in ACN, referred to as "ISTD mix", was added into extracts after SPE process. $^{13}C_{18}$-Zearalenone ($^{13}C_{18}$-ZEN), diluted with ACN to a final concentration of 100 ng/mL, was spiked into the sample before any sample preparation step. Concentrations of internal standards in samples are summarized in Table S2. All stock solutions and mixtures, including internal standards, were stored at -20 °C.

Pooled plasma and pooled serum used for method development and validation were purchased from Innovative Research (IPLAWBLIH) (Novi, MI, USA, https://www.innov-research.com/), and serum from Sigma-Aldrich (H4522, Sigma-Aldrich, Germany), respectively. *Helix pomatia* β-glucuronidase/arylsulfatase mixture was from Roche Diagnostics (27366-unit β-glucuronidase/mL, Vienna). Ammonium acetate (NH_4AC) was purchased from Sigma-Aldrich (Vienna). LC-MS grade methanol (MeOH) and acetonitrile (ACN) were purchased from Honeywell (Honeywell Austria Gmbh, Vienna), and LC-MS grade water (H_2O) was purchased from VWR (VWR International GmbH, Vienna). 96-well plates (2mL) and sealing mates were purchased from Waters Corporation (Vienna).

Sample Preparation

Definition of non-spiked, prespiked and postspiked samples

For pe-spiked samples, standards were added before sample preparation (i.e., dilution, enzyme hydrolysis, and the SPE process), whereas post-spiked samples were spiked with the STD mix after completing the SPE process. Non-spiked samples were pooled urine, plasma, or serum without adding the STD mix. Detailed descriptions for the used workflows can be found below.

Pre-spiking process

A volume of 20 mL pooled urine, 14 mL plasma, or 2 mL serum were mixed with the prepared STD mix, in an appropriate ratio to yield concentration levels presented in Table S14. The volume ratio of sample to the STD mix was >1:20 to avoid protein precipitation due to addition of organic solvents. The pre-spiked samples were vortexed for 3 mins at 1000 rpm, aliquoted and stored at -20 °C until the day of use.

SPE process

Based on results presented in our previous study (Gu et al., 2023), 400 µL of samples (H_2O for procedural blanks, non-spiked urine, plasma, and serum, or pre-spiked samples, see above) were mixed with 4 µL of $^{13}C_{18}$-ZEN (100 ng/mL) and diluted with 396 µL of phosphate-buffered saline (200 mM, PBS, pH 7.4), resulting in a final volume of 800 µL. The final concentrations of $^{13}C_{18}$-ZEN in samples are presented in Table S2. Our recently developed SPE clean-up method in 96-well plates (Gu et al., 2023) was used to extract the analytes from human samples using the following steps. First, columns were conditioned with 1 mL of MeOH, followed by 1 mL of H_2O. The diluted samples (see above 800 µL) were loaded on the SPE plates using multichannel pipettes and washed with 2x1 mL of H_2O. Analytes were eluted using 2x200 µL of MeOH. Then, 4 µL of the ISTD mix were added and extracts were diluted with 396 µL of H_2O, resulting in a final volume of 800 µL and a dilution factor of 2. Plates with the extracts were vortexed at 800 rpm for 3 mins before LC-MS analysis.

Enzyme hydrolysis for urine prior to SPE process

To assess the impact of deconjugation/enzyme hydrolysis on matrix effects, recovery and sensitivity, urine samples underwent enzymatic hydrolysis using a protocol adapted from Fareed et al. (2022). 625 µL of the *Helix pomatia* mixture containing β-glucuronidase and arylsulfatase were diluted with 4375 µL of 2.5M NH_4AC buffer (pH=5.0). Then, urine (400 µL) was mixed 4 µL of $^{13}C_{18}$-ZEN (100 ng/mL), and 1596 µL of the diluted enzyme mixture were added resulting in a volume of 2 mL. Samples were incubated at 37 °C at 400 rpm for 16 h and cleaned up using SPE, as described above.

Post-spiking process

Post-spiked samples were obtained by mixing the SPE eluates of non-spiked samples (urine, hydrolyzed urine, plasma and serum) with the STD mix. Then, 4 µL of the ISTD mix was added and samples were diluted with 396 µL of H_2O.

Preparation of external solvent calibration standards

First, the solvent for diluting the standards was prepared by mixing 2970 µL of 50% MeOH (MeOH/H_2O, 1/1, v/v), 15 µL of $^{13}C_{18}$-ZEN (100 ng/mL) and 15 µL of the ISTD mix. The highest calibration level (Level 9) was prepared by mixing 20 µL of the STD mix with 380 µL of the dilution solution. Serial dilutions were carried out using the dilution solution to yield all other calibration levels (see Table S10).

Preparation of matrix-matched calibration

Matrix-matched calibration standards were prepared for urine and plasma individually. The sample matrix was prepared by merging two extracts of pooled samples following the SPE protocol described above resulting in 1.6 mL of extracted matrix. The highest calibration level (Level 7) was obtained by mixing 11 µL of the STD mix with 209 µL of the corresponding matrix. All other calibration levels were then prepared by serial dilution (see Table S12).

Feasibility estimation for 96-well plate SPE protocol

To assess the applicability of the method for 234 compounds, pooled urine and plasma were quickly evaluated for trueness (RE) and matrix effects (SSE) before method validation. Either urine or plasma samples were prespiked (n=4 each matrix) and postspiked (n=4 each matrix) at calibration level 4 (see Table S12). Sample processing of prespiked and postspiked samples was performed as described above. The RE was calculated using the ratio of peak area for prespiked to postspiking samples after subtraction of peak areas of the non-spiked sample, while SSE was estimated as the ratio of peak areas of the postspiked to neat solvent (Gu et al., 2023; Matuszewski et al., 2003).

UPLC-MS/MS Analysis

The chromatographic separation was based on our previously published methods (see Preindl et al. (2019) and Jamnik et al. (2022)). An Acquity HSS T3 reversed-phase column (1.8 µm, 2.1 mm × 100 mm, Waters, Vienna) equipped with a Van-Guard pre-column (1.8 µm, Waters, Vienna) was used together with a 1290 Infinity II UPLC system (Agilent, Vienna). The column temperature was set to 40 °C, injection volume was 5 µL, and needle wash was performed using a mix of methanol/acetonitrile/isopropanol/water (1/1/1/1, v/v/v/v). As described in Table S4, eluent A was 0.3 mM of ammonium fluoride in H_2O and eluent B was 100% ACN and chromatographic separation was performed using a flow rate of 0.4 mL/min. The gradient elution program was as follows: initial condition were 5% solvent B and were held constant from 0.00 min to 1.00 min. Eluent composition was modified stepwise with solvent B linearly increasing from 5% to 18% between 1.00 min and 1.80 min, followed by an increase from 18% to 35% B between 1.80 min and 4.20 min, from 35% to 48% B between 4.20 min and 13.00 min, from 48% to 90% B between 13.00 min and 15.80 min, and from 90% to 98% between 15.80 min and 15.81 min. The column was then flushed with 98% B from 15.81 min to 17.60 min, followed by equilibration of the column under initial condition using 5% B from 17.70 min to 20.00 min. The Agilent 1290 Infinity II was coupled with a QTrap 7500 (Sciex) mass spectrometer equipped with an electrospray ionization (ESI) source (Sciex). Fast polarity switching in scheduled multiple reaction monitoring (MRM) mode was utilized for most compounds, with MRM transitions and other MS parameters presented in Table S6.

Quality Control

System stability test (SST)

An in-house SST solution was used to assess instrument performance before analytical batches. The SST solution consisted of a diverse set of compounds, selected to cover a wide range of polarities. These compounds included caffeine, phenylalanine, roxithromycin, zearalenone, bisphenol A, genistein, and fipronil, and was prepared in 10% ACN (ACN/H^2O, 1/9, v/v). Concentrations of the SST compounds range from 2.5 ng/mL to 100 ng/mL (see Table S7) and LC-MS/MS parameters are provided in Table S8 and Table S9.

Calibration and blanks

External calibration standards were prepared at nine concentration levels. Concentrations were adjusted for each compound individually to reflect different assay sensitivity and spanned a wide range of concentrations (0.005 pg/mL to 5 µg/mL, see Table S10). Linear calibration curves were generated within the working range using 1/x weighting. The linear range of each calibration curve and the corresponding coefficient of determination (R^2) values are presented in Table S11. Process blanks (H_2O used as sample for SPE) and solvent blanks (50% MeOH in H_2O, v/v, 1/1) were included to control background noise, contamination and to assess carryover.

QC strategy for high-throughput measurements

To ensure data reliability, three types of quality control (QC) samples were included in the analysis of the cohort of the YPOPS urine samples analyzed in the study. All 200 urine samples were pooled resulting in a pooled QC sample (referred to as "non-spiked QC"). Aliquots of the pooled sample were then used to create pre-spiked QC samples and post-spiked QC samples (see section above for details). Pre-spiked QC samples were used to assess the recovery of the SPE in this batch, whereas post-spiked QC samples were used to monitor matrix effects. Furthermore, internal standards were used as internal QCs. Process blanks, solvent blank, external calibration standards, and QC samples were analyzed before and after each batch of 20-30 cohort urine samples from the YPOPS study. Urine sample concentrations were blank-corrected by subtracting the mean concentration of each compound found in the process blanks (n=3).

Statistics for time trends analysis between concentrations and gestation weeks

Concentration trends of detected analytes over gestational weeks were analyzed using a Linear Mixed-Effects Model (R lmer function, version 4.5.0). Random effects were included for participants, which enabled the model to capture individual differences in the concentration trends across weeks of gestation (Brown, 2021). Fifty compounds were detected in more than 50% of the 200 urine samples and were selected for analyzing exposure dynamics. Prior to data normalization, non-detected concentrations were imputed as zero, and values below the limit of quantitation (LOQ) were imputed as half LOQ. All concentrations were then adjusted by adding 0.00001 before log 10-transformation. The Benjamini-Hochberg False Discovery Rate (FDR) was applied to control for false positives (R p.adjust function, version 4.5.0).

In-house Validation

The validation was conducted following the European Commission Decision (EC) No. 2021/808 (Commission, 2021), evaluating linearity, selectivity, matrix effects (assessed as signal suppression and enhancement, SSE), trueness (expressed as extraction recovery, RE), intermediate precision (inter-day precision, RSDR), repeatability (intra-day precision, RSDr), limit of detection (LOD), and limit of quantification (LOQ). Validation experiments were performed over three different days, with independent sample preparation, calibration, and measurements performed for each batch. Linearity was assessed by determining the R^2 for the external solvent calibration curves and through visual inspection of the calibration curves within the working range for all analytes. To evaluate selectivity, non-spiked pooled urine and plasma samples were also visually

inspected for potentially interfering peaks. Matrix effects and retention time shifts were assed using matrix-matched calibration for urine and plasma and at seven concentration levels (Table S12). SSE was determined as the ratio of the slopes of the matrix-matched calibration and slopes of calibration curves in pure solvent and was calculated across three independent batches (Matuszewski et al., 2003). The RE was calculated as the ratio of measured concentrations and added concentrations in pre-spiked urine and plasma samples, and was determined using matrix-matched calibration. Two spiking levels, approximately 3× and 30× LOQ values, were used for all 234 analytes (n=3 per spiking level and matrix). Measured concentrations were blank-corrected and concentrations found in the corresponding non-spiked urine or plasma samples (n=4) were subtracted. Concentrations of all spiking levels are provided in Table S14. RSDR was determined as the coefficient of variation (CV) of the RE obtained from three validation batches (n = 9 per spiking level and sample matrix). RSDr was determined by calculating the CV values within a single validation batch (n = 9 per spiking level and sample matrix).

LOD and LOQ were calculated based on the method described by Eurachem Guide (Cantwell, 2025). Three replicates of post-spiked urine/plasma with low concentrations were repeatedly analyzed (m = 9, see Table S13). For compounds without background in the non-spiked samples, LOD and LOQ were calculated based on a signal-to-noise ratio (S/N) of three and ten. For compounds with background the LOD and LOQ values were estimated by standard deviation of measured concentrations (s0). The measured concentrations were subtracted by concentrations found in the non-spiked urine/plasma/hydrolyzed urine for compounds with low background. The standard deviation of s0, divided by the square root of the number of sample replicates (n=1) and process blank replicates (nb=3) in routine analysis, was multiplied by 3 and 10 to determine the LOD and LOQ values, respectively. If the background was higher than 50% of the spiked concentration, blank subtraction was not applied (see scheme below).

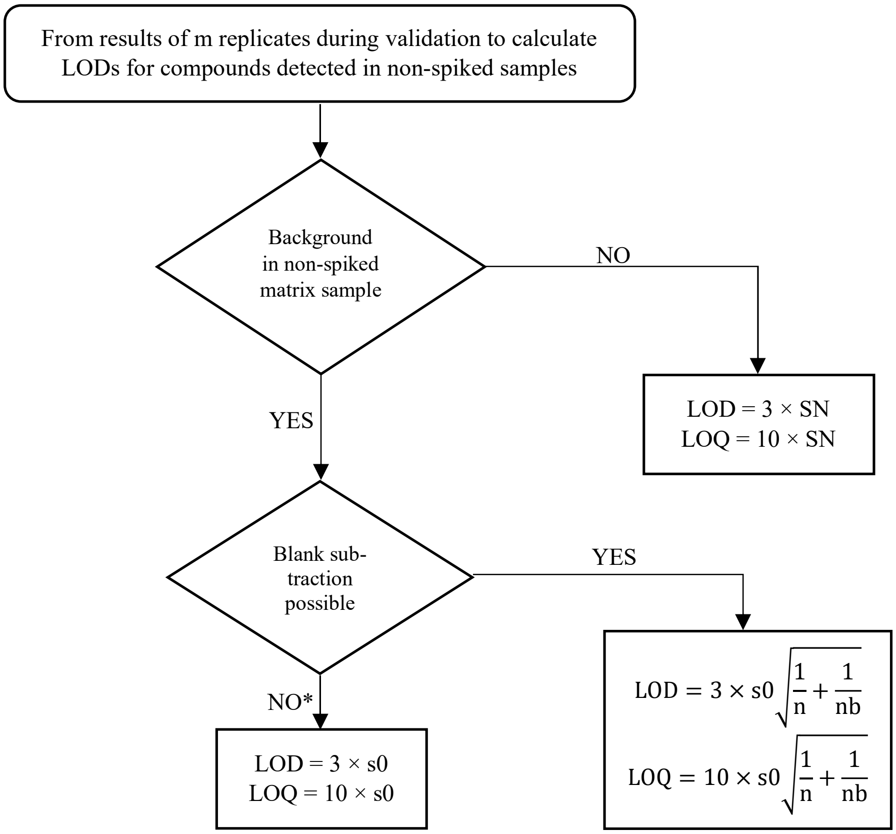

*Concentrations found in non-spiked samples were over 50% of the spiked levels.

Quick Validation for Serum and Enzyme Hydrolyzed Urine

To assess the applicability of the method to different human matrices, pooled serum was evaluated for trueness, repeatability, and matrix effects. For this purpose, samples were spiked at a single concentration of 30× LOQ ($n = 9$; concentrations provided in Table S14). RE, RSDr and SSE were calculated using the same approach as described above for pooled urine and plasma samples. The pre-spiked (n=9) and post-spiked (n=3) serum samples were prepared as described before. To assess the influence of enzyme hydrolysis on method performance, deglucuronidation and desulfation were carried out on urine samples before sample extraction using SPE process (see above) and trueness, repeatability, matrix effects, and sensitivity were evaluated. Non-spiked (n=4), pre-spiked (n=3) and post-spiked (n=3) urine samples were prepared and spiking levels are presented in Table S14. Repeated measurements of three replicates of post-spiked urine at low concentrations ($n = 9$ total, see Table S20) were analyzed to determine LOD and LOQ values.

Assessment for Efficacy and Environmental Impact

Sustainability of laboratory work is of emerging interest and in particular reduction of energy consumption, single-use lab ware and the use of (harmful) organic solvents is a critical factor in the design of analytical workflows today (Nowak et al., 2021). Sample extraction is a critical step to reduce avoidable solvent consumption and reduction scales with the number of samples analyzed. Especially for high-throughput assays optimization of extraction parameters can result in substantially reduced solvent requirements. Furthermore, total laboratory time requirements for (manual) sample extraction steps present a significant time- and cost factor when preforming large-scale measurements. To fully assess the sustainability of our method in terms of solvent consumption and time requirements, we compared predictions for this workflow with other typical workflows in human biomonitoring and exposomics (see Table S17).

Results and Discussion

Initial performance estimation for 96-well plate-based SPE

In total, 68% of all compounds in urine and 78% of the analytes in plasma could be extracted with RE in the range of 60% - 140%, and 84% and 81% of compounds showed SSE between 60%-140% in urine and plasma samples, respectively (see **Figure S1**).

Method performance for serum and enzymatically hydrolyzed urine

Figure S3 summarizes the method performance in serum and hydrolyzed urine. RE in the range of 42%-134% was observed for 62% and 64% of compounds in serum and hydrolyzed urine, respectively, with corresponding RSDr below 37%. Evaluation of matrix effects revealed that 63% and 50% of analytes in serum and hydrolyzed urine, respectively, exhibited SSE between 60% and 140%. Similar to plasma, serum showed distinct trends for compounds with SSE values outside the 60%-140% range. Some polar compounds, typically with a logP below 3.0, exhibited matrix enhancement (SSE > 140%) while some nonpolar chemicals displayed matrix suppression (SSE < 60%). For hydrolyzed urine the behavior was similar to the non-hydrolyzed urine. Furthermore, the method demonstrated good sensitivity in serum, with 74% of analytes achieving LODs below 1.0 ng/mL, and 3% with LODs below 0.001 ng/mL. Detailed recovery and intra-day RSD data can be found in Table S14, whereas LOD, LOQ, and SSE in serum and hydrolyzed urine are presetned in Table S20.

Supplementary Figures

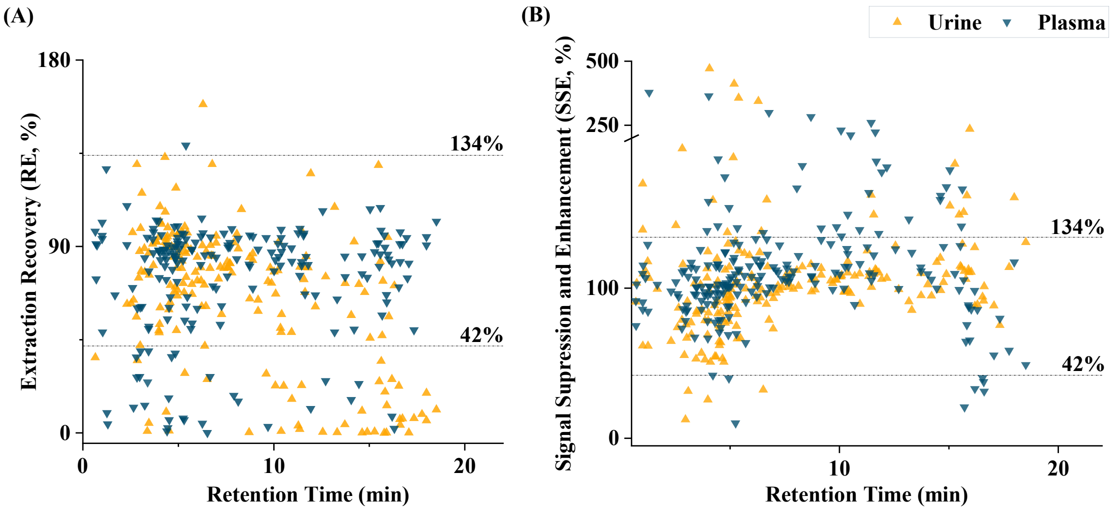

Figure S1. (A) Extraction recovery (RE, %) (B) and signal suppression and enhancement (SSE, %) for 234 compounds in pooled urine and plasma samples during a first feasibility study.

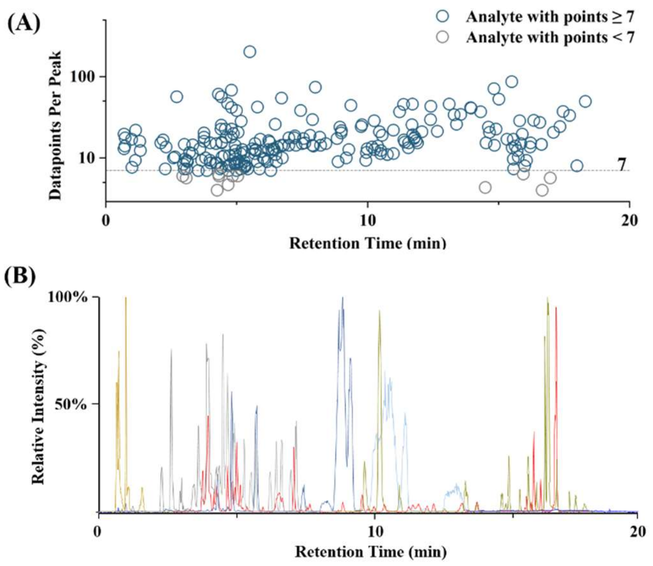

Figure S2. Distribution of targeted analytes over the retention time range of the LC-method (RT, min), including (A) effect of coelution and chromatographic separation on the mean number of datapoints per peak in post-spiked pooled urine (n=3), and (B) chromatographic peaks of all analytes in MeOH/H_2O (1/1, v/v).

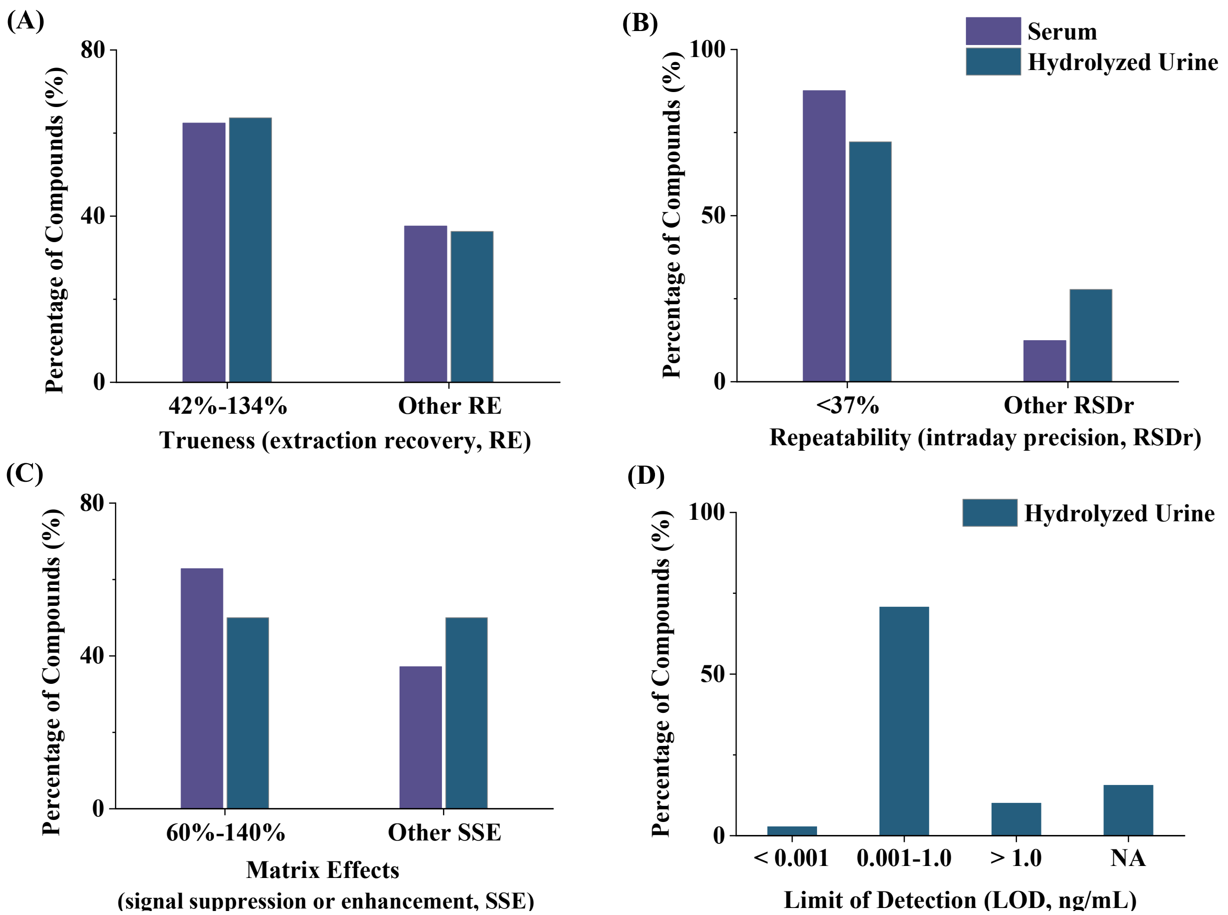

Figure S3. Percentage of compounds in serum and/or hydrolyzed urine with extraction recovery (RE, A), intraday precision (RSDr, B), signal suppression/enhancement (SSE, C) and limit of detection (LOD, D) with specified ranges. "NA" indicates not applicable data.

Reference

Braun, D., Abia, W. A., Šarkanj, B., Sulyok, M., Waldhoer, T., Erber, A. C., Krska, R., Turner, P. C., Marko, D., Ezekiel, C. N., & Warth, B. (2022). Mycotoxin-mixture assessment in mother-infant pairs in Nigeria: From mothers' meal to infants' urine. *Chemosphere*, *287*(Pt 2), 132226. https://doi.org/10.1016/j.chemosphere.2021.132226

Brown, V. A. (2021). An introduction to linear mixed-effects modeling in R. *Advances in Methods and Practices in Psychological Science*, *4*(1), 2515245920960351.

Caballero-Casero, N., Castro, G., Bastiaensen, M., Gys, C., van Larebeke, N., Schoeters, G., & Covaci, A. (2021). Identification of chemicals of emerging concern in urine of Flemish adolescents using a new suspect screening workflow for LC-QTOF-MS. *Chemosphere*, *280*, 130683. https://doi.org/10.1016/j.chemosphere.2021.130683

Eurachem Guide: The Fitness for Purpose of Analytical Methods: A Laboratory Guide to Method Validation and Related Topics: Third edition (2025), (2025). http://www.eurachem.org

Commission, E. (2021). Commission Implementing Regulation (EU) 2021/808 of 22 March 2021 on the performance of analytical methods for residues of pharmacologically active substances used in food-producing animals and on the interpretation of results as well as on the methods to be used for sampling and repealing Decisions 2002/657/EC and 98/179/EC. *Off. J. Eur. Union*, *180*, 84-109.

Fareed, Y., Braun, D., Flasch, M., Globisch, D., & Warth, B. (2022). A broad, exposome-type evaluation of xenobiotic phase II biotransformation in human biofluids by LC-MS/MS. *Exposome*, *2*(1), osac008. https://doi.org/10.1093/exposome/osac008

Frederiksen, H., Nielsen, O., Koch, H. M., Skakkebaek, N. E., Juul, A., Jørgensen, N., & Andersson, A.-M. (2020). Changes in urinary excretion of phthalates, phthalate substitutes, bisphenols and other polychlorinated and phenolic substances in young Danish men; 2009–2017. *International journal of hygiene and environmental health*, *223*(1), 93-105.

Frigerio, G., Campo, L., Mercadante, R., Santos, P. M., Missineo, P., Polledri, E., & Fustinoni, S. (2020). Development and validation of a liquid chromatography/tandem mass spectrometry method to quantify metabolites of phthalates, including di-2-ethylhexyl terephthalate (DEHTP) and bisphenol A, in human urine. *Rapid Communications in Mass Spectrometry*, *34*(13), e8796.

Gao, D., Yang, J., Bekele, T. G., Zhao, S., Zhao, H., Li, J., Wang, M., & Zhao, H. (2020). Organophosphate esters in human serum in Bohai Bay, North China. *Environmental Science and Pollution Research*, *27*, 2721-2729.

Gu, Y., Peach, J. T., & Warth, B. (2023). Sample preparation strategies for mass spectrometry analysis in human exposome research: Current status and future perspectives. *TrAC Trends in Analytical Chemistry*, 117151.

Guo, C., Zhao, X., Jin, J., Wang, L., Tan, D., Chen, J., & Ni, Y. (2021). The dose effect of dansyl chloride on the derivative products of bisphenols and its application for the determination of bisphenols in human serum by high-performance liquid chromatography–tandem mass spectrometry. *Journal of Separation Science*, *44*(16), 3052-3060.

Guo, J., Wu, C., Zhang, J., Jiang, S., Lv, S., Lu, D., Qi, X., Feng, C., Liang, W., & Chang, X. (2019). Anthropometric measures at age 3 years in associations with prenatal and postnatal exposures to chlorophenols. *Chemosphere*, *228*, 204-211.

Gys, C., Bastiaensen, M., Malarvannan, G., Bamai, Y. A., Araki, A., & Covaci, A. (2021). Short-term variability of bisphenols in spot, morning void and 24-hour urine samples. *Environmental Pollution*, *268*, 115747.

Hossain, M. Z., Feuerstein, M. L., Gu, Y., & Warth, B. (2024). Scaling up a targeted exposome LC-MS/MS biomonitoring method by incorporating veterinary drugs and pesticides. *Analytical and Bioanalytical Chemistry*, 1-14.

Jala, A., Varghese, B., Dutta, R., Adela, R., & Borkar, R. M. (2022). Levels of parabens and bisphenols in personal care products and urinary concentrations in Indian young adult women: implications for human exposure and health risk assessment. *Chemosphere*, *297*, 134028.

Jamnik, T., Flasch, M., Braun, D., Fareed, Y., Wasinger, D., Seki, D., Berry, D., Berger, A., Wisgrill, L., & Warth, B. (2022). Next-generation biomonitoring of the early-life chemical exposome in neonatal and infant development. *Nat Commun*, *13*(1), 2653. https://doi.org/10.1038/s41467-022-30204-y

Li, Y., Li, D., Chen, J., Zhang, S., Fu, Y., Wang, N., Liu, Y., & Zhang, B. (2020). Presence of organophosphate esters in plasma of patients with hypertension in Hubei Province, China. *Environmental Science and Pollution Research*, *27*, 24059-24069.

Matuszewski, B. K., Constanzer, M., & Chavez-Eng, C. (2003). Strategies for the assessment of matrix effect in quantitative bioanalytical methods based on HPLC− MS/MS. *Analytical Chemistry*, *75*(13), 3019-3030.

Nowak, P. M., Wietecha-Posłuszny, R., & Pawliszyn, J. (2021). White analytical chemistry: an approach to reconcile the principles of green analytical chemistry and functionality. *TrAC Trends in Analytical Chemistry*, *138*, 116223.

Preindl, K., Braun, D., Aichinger, G., Sieri, S., Fang, M., Marko, D., & Warth, B. (2019). A Generic Liquid Chromatography-Tandem Mass Spectrometry Exposome Method for the Determination of Xenoestrogens in Biological Matrices. *Anal Chem*, *91*(17), 11334-11342. https://doi.org/10.1021/acs.analchem.9b02446

Siddique, S., Harris, S. A., Kosarac, I., Latifovic, L., & Kubwabo, C. (2020). Urinary metabolites of organophosphate esters in women and their relationship with serum lipids: An exploratory analysis. *Environmental Pollution*, *263*, 114110.

Varghese, B., Jala, A., Das, P., Borkar, R. M., & Adela, R. (2022). Estimation of parabens and bisphenols in maternal products and urinary concentrations in Indian pregnant women: daily intake and health risk assessment. *Environmental Science and Pollution Research*, 1-14.
